# Salivary Redox Biomarkers as a Non-Invasive Research Framework for Exploring Redox-Related Cardiac Electrical Vulnerability in Sudden Unexplained Cardiac Death: A Mechanistic and Narrative Review

**DOI:** 10.3390/medicina62020394

**Published:** 2026-02-18

**Authors:** Ahmed Adel Mansour Kamar, Ioannis Mavroudis, Alin Ciobica, Diana Gheban

**Affiliations:** 1 Medical Department, Gulf of Suez Petroleum Company (GUPCO)—Cairo Office, Cairo 11511, Egypt; 2 Department of Orthopedics and Traumatology, Clinical Recovery Hospital (Recuperare), 700661 Iasi, Romania; 3 Doctoral School, Faculty of Biology, “Alexandru Ioan Cuza” University of Iasi, 700505 Iasi, Romania; 4 Neuroscience Department, Leeds Teaching Hospitals NHS Trust, Leeds LS1 3EX, UK; 5 Laboratory of Neuropathology & Electron Microscopy, School of Medicine, Faculty of Health Sciences, Aristotle University of Thessaloniki, 54124 Thessaloniki, Greece; 6 Department of Biology, Faculty of Biology, “Alexandru Ioan Cuza” University of Iasi, 700505 Iasi, Romania; 7 “Ioan Hăulică” Institute, “Apollonia” University, 700511 Iasi, Romania; 8 Biomedical Research Group, “Olga Necrasov” Center, Romanian Academy, 700481 Iasi, Romania; 9 Academy of Romanian Scientists, Ilfov No. 3, 030167 Bucharest, Romania; 10 Faculty of Medicine and Biomedical Sciences, Apollonia University, 700511 Iasi, Romania

**Keywords:** sudden unexplained cardiac death, oxidative stress, salivary biomarkers, superoxide dismutase, catalase, glutathione peroxidase, malondialdehyde, total antioxidant capacity, Salivary Redoxome, non-invasive diagnostics, precision cardiology

## Abstract

Sudden unexplained cardiac death (SUCD) is unpredictable, causing major emotional, economic, and productivity loss. In young, apparently healthy individuals, it remains one of the most challenging causes of mortality to understand mechanistically, and no validated molecular biomarkers are currently available to support investigation of subclinical cardiac electrical vulnerability. Conventional clinical assessment tools such as electrocardiography, echocardiography, and genetic testing often fail to detect early molecular disturbances that precede electrical or structural cardiac abnormalities. Recent evidence suggests that oxidative stress and redox imbalance play a crucial mechanistic role in cardiac electrical instability, modulating ion channel function, calcium handling, mitochondrial signaling, and intercellular coupling. This literature review explores the emerging role of salivary redox biomarkers as a non-invasive research framework for exploring redox-related mechanisms relevant to cardiac electrophysiology, introducing the concept of a “Salivary Redoxome”—an integrated oxidative–antioxidative profile measurable in saliva that may reflect systemic redox homeostasis in an exploratory context, without implying myocardial specificity. Key antioxidant enzymes such as superoxide dismutase (SOD), catalase (CAT), and glutathione peroxidase (GPx), together with oxidative damage indices such as malondialdehyde (MDA) and total antioxidant capacity (TAC), are discussed for their theoretical mechanistic relevance, biological plausibility, and current limitations. Methodological considerations, sources of pre-analytical variability, and challenges related to biomarker specificity and validation are also addressed to contextualize the current evidence base. At present, no direct clinical evidence links salivary oxidative stress markers to sudden unexplained cardiac death or to electrophysiological arrhythmic risk, and their proposed relevance is therefore exploratory and hypothesis-generating. This review positions salivary redox profiling as a research approach rather than a clinical screening, predictive, or preventive tool, and outlines a future research agenda aimed at systematic validation in well-designed prospective studies.

## 1. Introduction

Sudden cardiac death (SCD) refers to an unexpected death due to cardiac causes occurring within a short time period, generally within one hour of symptom onset, or within 24 h of last being seen well. In younger individuals, SCD may result from structural heart disease, inherited cardiomyopathies, or primary electrical disorders. SUCD represents a distinct subset of SCD in which no definitive cause is identified despite comprehensive investigation, including detailed autopsy, histopathology, toxicological analysis, and, where available, molecular autopsy. Importantly, SUCD does not imply absence of underlying vulnerability but rather reflects current diagnostic limitations in identifying subtle electrical or molecular abnormalities [1,2,3].

SCD in young healthy individuals 25–40 years of age represents a devastating, poorly understood phenomenon worldwide. A substantial proportion of these cases estimated at up to 30–40% remains unexplained and continues to challenge clinicians and researchers worldwide. Although its overall incidence is relatively low compared to adult sudden cardiac death—typically occurring in individuals older than 40 years and mainly attributable to acquired cardiovascular disease, the societal and emotional impact of losing apparently healthy adolescents and young adults to unexpected fatal arrhythmias is profound. Despite advances in cardiac imaging, electrophysiological testing, and molecular genetics, this diagnostic gap highlights the presence of subtle, preclinical molecular alterations that are not captured by current clinical assessment approaches [1,3,4].

Growing evidence implicates oxidative stress and redox imbalance as central contributors to cardiac electrical instability. Reactive oxygen and nitrogen species influence multiple targets involved in cardiac excitability, including ion channels, calcium-handling proteins, and mitochondrial pathways. Under physiological conditions, the heart maintains a delicate balance between pro-oxidant and antioxidant mechanisms. Disruption of this equilibrium can induce electrophysiological heterogeneity, prolong repolarization, and trigger arrhythmogenic afterdepolarizations, even in the absence of an overt structural pathology. Detecting such redox alterations could provide a molecular research tool for exploring cardiac vulnerability in individuals who appear healthy [5,6,7,8,9].

Given its accessibility, saliva has recently emerged as a promising biological fluid for non-invasive biomarker research [7,10,11]. Salivary components reflect systemic biochemical status through capillary filtration and glandular secretion, offering a practical and ethically acceptable method for repeated sampling [7]. This review explores the scientific and mechanistic basis for using salivary redox biomarkers as potential indicators of redox-related cardiac vulnerability and outlines the pathway toward future validation and translational research, rather than immediate clinical implementation.

## 2. Aim of the Literature Review

The aim of this literature review is to explore and critically synthesize current scientific evidence linking oxidative stress and redox imbalance to the pathophysiology of sudden unexplained cardiac death and sudden cardiac death in young healthy individuals and athletes. This work is designed as a provisional and mechanistic study to support future hypothesis-driven research and systematic biomarker validation studies on the proposed concept of salivary redox biomarkers (“Salivary Redoxome”) as a non-invasive research approach to study early cardiac electrical vulnerability.

The specific objectives of this review are to:

- Clarify the molecular and electrophysiological mechanisms through which oxidative stress contributes to cardiac electrical instability and arrhythmogenesis;
- Assess the current state of knowledge regarding oxidative and antioxidative biomarkers—particularly those measurable in saliva, such as SOD, CAT, GPx, MDA, and TAC;
- Identify the limitations, inconsistencies, and research gaps in existing studies that hinder the translation of salivary redox markers into clinically meaningful and reproducible research biomarkers;
- Describe the steps for future clinical validation studies and to assess how the Salivary redoxome may help to explore and better understand early cardiac electrical vulnerability in young individuals.

Ultimately, this literature review aims to provide a scientific and conceptual basis for developing a non-invasive and cost-effective biomarker strategy capable of supporting future hypothesis-driven research on redox-related cardiac vulnerability of SUCD in young individuals and athletes.

### Research Question

Can oxidative stress biomarkers provide a mechanistic research framework for exploring early redox-related cardiac vulnerability in the context of sudden unexplained cardiac death?

## 3. Methodology

A literature search was performed in PubMed, Scopus, Web of Science, and Google Scholar covering publications from January 2000 to March 2025. After removal of duplicates, approximately 80 records were screened at the title and abstract level. Of these, 34 articles were reviewed in full text, and 27 studies were included based on relevance to the review objectives. Literature screening, study selection, and data extraction were performed. This approach was adopted to ensure conceptual consistency in the qualitative integration of mechanistic evidence within a narrative review framework. This work was conducted as a narrative mechanistic literature review; therefore, no formal systematic review protocol or risk-of-bias assessment was applied. Boolean operators (AND/OR) were used to combine keywords related to oxidative stress, redox imbalance, cardiac electrophysiology, arrhythmia, sudden cardiac death, and salivary biomarkers, including superoxide dismutase, catalase, glutathione peroxidase, malondialdehyde, and total antioxidant capacity. Studies were prioritized based on mechanistic validity and translational relevance, defined as a clear biological link between redox imbalance, cardiac electrophysiological dysfunction, and arrhythmogenic pathways. Studies were included if they provided mechanistic, translational, or clinical evidence related to oxidative stress and cardiac electrical instability; cellular, animal, and human investigations were all considered. Studies were excluded if they were unrelated to cardiovascular mechanisms or focused exclusively on local oral pathology without systemic relevance. Extracted data were analyzed qualitatively to identify molecular mechanisms, methodological limitations, and potential research gaps that could inform future research validation studies. As the work relies exclusively on published data, ethical approval was not required.

## 4. Scientific Background and Clinical Perspectives

Sudden death in young individuals can arise from many causes and is often termed sudden “unexpected” death in the young (SUDY). When it is due to a cardiac cause, it is classified as SCD, which includes multiple mechanisms such as cardiomyopathies, ischemic heart disease, trauma, viral infection, stress or substance-related effects. When no cause can be identified after appropriate evaluation, the event is classified as SUCD. SCD may be the first manifestation of concealed disease in asymptomatic individuals and the diagnosis is often made only through a detailed postmortem examination. So, in young individuals, SUCD constitutes a subset of SCD in which no structural, toxicological, or genetic cause is identified despite comprehensive investigation [12,13,14,15].

### 4.1. Epidemiology

In the European Union, SCD accounts for approximately a quarter of a million deaths are reported annually and 4 million worldwide, representing a major public health problem [2].

**Figure 1:**
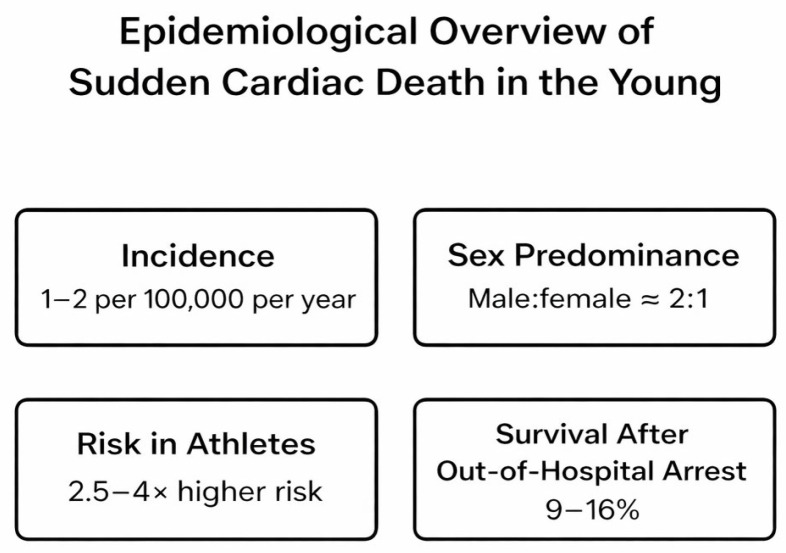
Epidemiological overview of sudden cardiac death in young adults aged 25–40 years. The figure summarizes key epidemiological indicators: an estimated incidence of 1–2 cases per 100,000 persons per year, a male-to-female predominance of approximately 2:1, a 2.5–4-fold higher risk among athletes compared with non-athletes, and a 9–16% survival rate following out-of-hospital cardiac arrest. As reported in population-based and registry studies [1,3,4,15]. This figure is an original conceptual illustration created by the authors; AI-based software was used only to assist with graphic visualization.

Sudden cardiac death represents an important cause of unexpected death. SCD in young adults, apparently healthy individuals, remains a rare yet deeply impactful phenomenon within global cardiovascular mortality. As many as half of sudden cardiac deaths are unexpected, occurring without preceding symptoms or signs of cardiac disease [16]. Contemporary data indicate that the incidence of sudden cardiac death among individuals 25–40 years of age in “European populations” ranges between 1 and 2 cases per 100,000 persons per year [1,4] (Figure 1), with considerable regional and demographic variability.

Although recent advances in imaging, genetics, and emergency care have improved the diagnosis and survival of some explained cases of SCD in Europe, these tools appear to have had limited impact on sudden unexplained cardiac death. Young individuals who experience SUCD often show completely normal findings before the event, and the cause remains unknown even after a full investigation. Recent North American trends demonstrate a gradual increase in SCD incidence among younger adults, particularly those aged 25–44 years. This rise has been associated with multifactorial influences, including lifestyle factors, metabolic stress, subclinical cardiomyopathies, and environmental contributors such as air pollution and psychosocial stress, as suggested in broader SCD datasets rather than SUCD-specific analyses [4,15,17].

Despite improved autopsy techniques and the incorporation of molecular autopsy into forensic protocols, a substantial proportion—estimated at 30% to 40%—of sudden deaths in the young remain unexplained, lacking identifiable structural, toxicological, or genetic causes [1,3]. This unexplained subset represents a critical target population for biomarker-based research approaches and future validation studies.

Epidemiological observations consistently reveal a male predominance, with male-to-female ratios approximating 2:1 across most cohorts, possibly reflecting sex-related differences in myocardial repolarization, hormonal modulation of ion channels, and exposure to physical stressors. Geographic and ethnic disparities are also evident, with higher incidence rates observed in specific minority and low-resource populations.

In athletes and individuals engaged in high-intensity physical activity, the risk of sudden cardiac arrest is elevated—approximately 2.5- to 4-fold greater than in age-matched non-athletic counterparts [4,15]—underscoring the limitations of conventional pre-participation screening. Survival after out-of-hospital cardiac arrest in young adults remains disappointingly low, typically ranging from 9% to 16% worldwide. Collectively, these epidemiological patterns highlight the persistent diagnostic blind spot preceding SUCD and reinforce the necessity for molecular-level research approaches aimed at exploring preclinical electrical and redox instability [18].

### 4.2. Mechanisms of Sudden Cardiac Death

**Figure 2:**
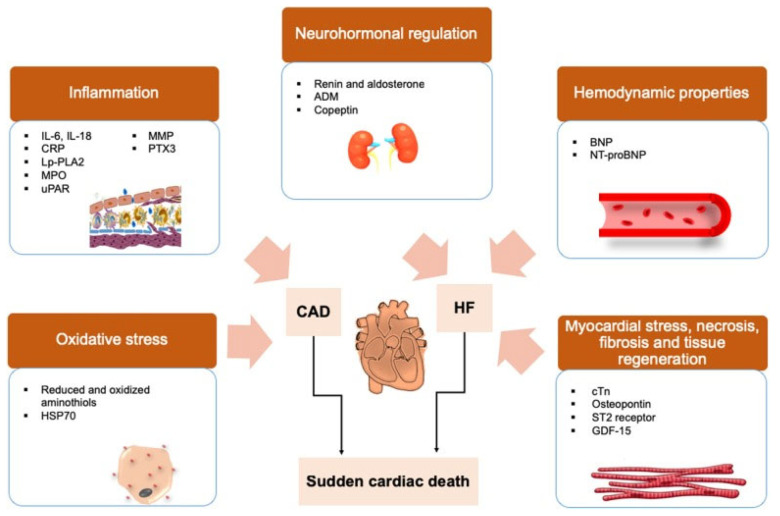
Biomarker pathways involved in sudden cardiac death. Protein biomarkers for research-based assessment of mechanisms related to SCD. Surrogate biomarkers that reflect the development of oxidative stress and inflammation are associated with coronary artery diseases (CAD). As biomarkers that reflect the neurohormonal regulation process, hemodynamic properties and myocardial stress are often associated with heart failure (HF). Adapted from Osman J. et al. [3], distributed under the Creative Commons Attribution (CC BY) license.

SCD results from the abrupt loss of effective cardiac output, most often due to malignant ventricular arrhythmias such as ventricular tachycardia or ventricular fibrillation. These arrhythmias arise from disturbances in myocardial excitability, conduction, and repolarization that promote re-entry circuits, triggered activity, or abnormal automaticity [5,6] (Figure 2).

SUCD is a multifactorial phenomenon rather than the result of a single linear pathogenic pathway. Although oxidative stress and redox imbalance contribute to cardiac electrical instability, they act as modulatory and amplifying factors within a broader network that includes genetic ion-channelopathies, subtle or preclinical cardiomyopathies, autonomic nervous system imbalance, and acute environmental or lifestyle triggers. Factors such as intense physical exertion, psychological stress, sleep disturbances, air pollution, and nutritional antioxidant status may influence systemic redox homeostasis and lower the arrhythmic threshold in susceptible individuals. Therefore, oxidative stress should not be interpreted as the sole cause of SUCD, but as a measurable component of an integrated pathophysiological framework that reflects cumulative vulnerability rather than deterministic causation [3,5,6,8,18].

In younger individuals and athletes, SCD typically occurs in the absence of coronary artery disease and is associated with inherited or acquired electrical and structural disorders, including ion-channelopathies—such as long QT syndrome, Brugada syndrome, and catecholaminergic polymorphic ventricular tachycardia—and cardiomyopathies, particularly hypertrophic and arrhythmogenic forms. Despite these recognized entities, a considerable proportion of cases remain unexplained at autopsy or molecular analysis, classified as SUCD [3,6,8,16].

At the cellular level, oxidative stress and redox imbalance have emerged as key contributors to arrhythmogenic remodeling. Reactive oxygen species (ROS) modify ion-channel function, disrupt calcium handling, and impair mitochondrial energy metabolism, leading to electrical heterogeneity and instability even in structurally normal hearts Oxidative activation of Ca^2+^/calmodulin-dependent protein kinase II (CaMKII) further amplifies calcium leakage and afterdepolarizations, establishing a substrate conducive to lethal ventricular arrhythmias [5,9,18,19,20].

SCD therefore represents the final common pathway of diverse insults that converge on oxidative and electrophysiological dysregulation of the myocardium, underscoring the rationale for investigating redox biomarkers as research correlates of electrical vulnerability [3,19,21].

### 4.3. Oxidative Stress and Cardiac Electrophysiology

Oxidative stress represents an imbalance between the production of ROS and the capacity of antioxidant defenses to neutralize them. In cardiac tissue, ROS generation arises from mitochondrial respiration, NADPH oxidases, xanthine oxidase, and uncoupled nitric oxide synthase [5,19]. Excessive ROS modifies key electrophysiological components, including sodium (Na^+^), calcium (Ca^2+^), and potassium (K^+^) channels, thereby disturbing action potential formation and propagation [6,8]. Oxidative modifications such as S-nitrosylation and S-glutathionylation alter ion channel gating, decrease sodium current amplitude, and prolong repolarization, increasing susceptibility to arrhythmia [5,6].

### 4.4. Molecular Pathways Linking Redox Imbalance to Arrhythmogenesis

Beyond direct channel modulation, oxidative stress affects calcium handling by altering the function of ryanodine receptors, sarcoplasmic reticulum Ca^2+^-ATPase, and CaMKII [5,6,9,22]. Oxidized CaMKII triggers abnormal calcium leakage and afterdepolarizations that can initiate lethal arrhythmias [5,8,9]. Mitochondrial dysfunction further amplifies ROS production, disrupting ATP generation and promoting electrical instability [6,9,19]. Together, these mechanisms may contribute to a self-sustaining cycle of redox dysregulation, energetic failure, and arrhythmogenic remodeling [5,9,22].

### 4.5. Salivary Redox Biomarkers: Composition and Systemic Reflection

Saliva contains numerous oxidative stress markers that may reflect systemic redox status. Antioxidant enzymes such as SOD, CAT, and GPx constitute the enzymatic defense line against ROS. SOD catalyzes the conversion of superoxide anion into hydrogen peroxide, which is then decomposed by CAT and GPx. Non-enzymatic antioxidants, including uric acid, glutathione, and ascorbic acid, contribute to TAC. MDA, a lipid peroxidation product, serves as an indicator of oxidative damage [7,19,23,24]. Evidence suggests that these salivary parameters correlate with plasma or serum equivalents, supporting saliva as a potential diagnostic matrix in a research context [10,20,24].

### 4.6. Evidence Supporting Systemic Cardiovascular Relevance of Salivary Biomarkers

The systemic relevance of salivary biomarkers in cardiovascular research is supported by studies demonstrating measurable salivary changes in established cardiac conditions. Salivary proteins and enzymes, including inflammatory and stress-related markers, have been shown to correlate with acute myocardial infarction and to complement electrocardiographic assessment, indicating that saliva can reflect systemic cardiovascular pathophysiology without implying cardiac tissue specificity [7,18].

These observations support the use of saliva as a non-invasive matrix for studying systemic biochemical environments relevant to cardiovascular vulnerability (Table 1). However, such findings do not establish organ-specific signaling and reinforce the need to interpret salivary biomarkers as indicators of global physiological states rather than direct surrogates of myocardial pathology.

### 4.7. Summary of Salivary Redox Biomarkers in Cardiovascular Research

**Figure 3:**
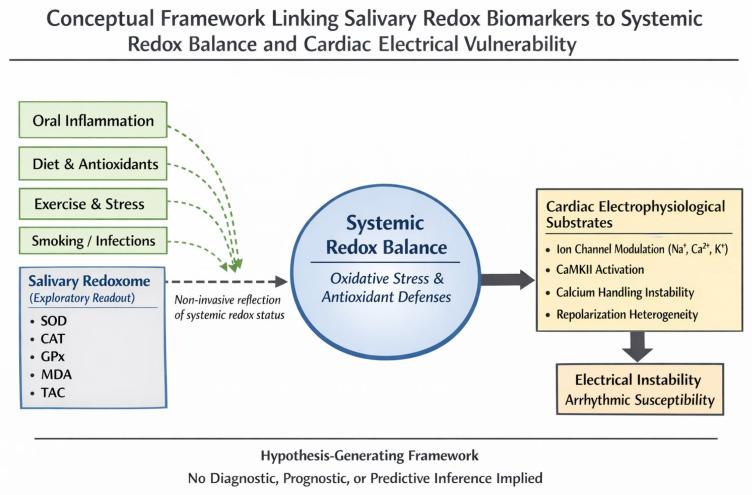
Conceptual framework linking salivary redox biomarkers to cardiac electrical vulnerability. Salivary redox biomarkers are shown as a non-invasive, exploratory reflection of systemic redox balance. Systemic redox imbalance may influence cardiac electrophysiological processes and contribute to electrical vulnerability. This figure is hypothesis-generating and is not intended for diagnostic or predictive use. Original Figure created by the authors.

Current research shows that salivary redox biomarkers can reflect the body’s overall oxidative balance and are altered in several cardiovascular and metabolic conditions linked to arrhythmia risk. Although no studies have yet directly connected salivary oxidative markers with sudden unexplained cardiac death, available evidence supports their biological relevance through effects on ion channels, calcium handling, and mitochondrial function. Because saliva collection is non-invasive and correlates with blood-based oxidative markers, salivary redox profiling represents a promising research-oriented approach. Nevertheless, factors such as lifestyle, physical activity, and sampling conditions can influence results, highlighting the need for standardized protocols and prospective validation before any clinical applicability can be considered [7,11,24]. This conceptual relationship is illustrated in Figure 3.

### 4.8. Analytical and Clinical Validity of Salivary Oxidative Markers

Quantifying salivary redox biomarkers requires standardized collection and storage procedures to avoid pre-analytical variability. Both spectrophotometric and ELISA-based assays have been used for measuring SOD, CAT, GPx, MDA, and TAC, demonstrating reasonable reproducibility [7,24,25]. Clinically, altered salivary oxidative profiles have been reported in cardiovascular, metabolic, and neurodegenerative diseases, indicating their broader systemic relevance [10,19,21,25]. Translating these observations into research applications for cardiac vulnerability assessment necessitates validation in young healthy cohorts, correlation with electrophysiological indices such as QT dispersion or heart rate variability, and longitudinal follow-up to link biomarker shifts with clinical outcomes [3,16].

### 4.9. Pre-Analytical and Physiological Confounders

Several factors influence salivary oxidative parameters, including circadian rhythm, diet, exercise, oral hygiene, and inflammatory conditions of the oral cavity. Controlling these variables is critical for biomarker reliability. Standardized sampling protocols—such as morning fasting collection, avoidance of physical exertion and smoking before sampling, and exclusion of participants with periodontal disease—are recommended for reproducibility and comparability across studies [7,24,25].

### 4.10. Emerging Technologies and Biosensor Innovations

Advances in microfluidics, nanomaterials, and electrochemical sensing have accelerated the development of portable salivary biosensors capable of real-time redox measurement. Integration of these devices with smartphone-based data acquisition could enable decentralized data collection in research settings [11,25]. The envisioned “Salivary Redoxome” platform would combine multiplex detection of enzymatic and non-enzymatic markers, applying machine-learning algorithms to explore redox profile patterns and support hypothesis generation related to SUCD vulnerability [11,21]. Such innovations align with ongoing research efforts in analytical and digital health technologies.

## 5. Discussion

Integrating the reviewed evidence, it becomes clear that oxidative stress serves as a mechanistic bridge between molecular changes and electrical vulnerability in SUCD [18]. While serum-based oxidative biomarkers have been extensively studied, saliva offers a more accessible medium for population-based and research-focused investigations, particularly in young individuals without overt disease. Importantly, these exploratory associations must not be interpreted as evidence of causality or predictive capability, but rather as hypothesis-generating observations that require rigorous prospective validation.

The correlation between salivary and systemic antioxidant status indicates that salivary biomarkers could serve as exploratory indicators of subclinical redox imbalance. For translational application, a structured validation pathway is essential. Initial pilot studies should establish normative salivary redox reference ranges in healthy young populations. Subsequent cross-sectional studies can compare individuals with benign ECG abnormalities or genetic variants predisposing to arrhythmia, while longitudinal designs can explore associative relationships with future cardiac events. A saliva-based redox test, if validated, could represent a low-cost research tool to support future investigational strategies which are typically associated with medical and societal costs, making this approach a potentially cost-efficient research framework. Also, saliva sampling poses minimal to no harm, making it an ethically robust approach suitable for young individuals, athletes, and research cohorts. Such a research-based approach could support future efforts aimed at improving scientific understanding of unexpected cardiac deaths in selected research populations, including athletes and high-stress professional groups such as military personnel and firefighters [15].

Importantly, because SUCD incidence varies across Europe, coordinated multicenter research initiatives are needed to harmonize biomarker protocols, build research biobanks of salivary samples linked with ECG and genetic data, and test cost-effectiveness across diverse populations. Collaboration among centers would help ensure methodological consistency and broader applicability within a research context, without implying clinical implementation.

## 6. Conceptual Nature and Current Limitations

All associations discussed in this review are exploratory and non-causal, and no predictive or clinical inference should be drawn in the absence of prospective validation. It is important to emphasize that the proposed use of salivary redox biomarkers for assessing redox-related cardiac electrical vulnerability in the context of sudden unexplained cardiac death remains conceptual and hypothesis-generating. To date, no prospective clinical studies have directly evaluated associations between salivary oxidative stress markers and established electrophysiological risk indicators such as QT dispersion, premature ventricular complex burden, or heart rate variability in healthy pediatric or young adult populations. In addition, the relatively low incidence of SUCD presents inherent challenges for population-level investigation and limits current prognostic applicability.

Salivary oxidative stress markers are influenced by factors such as diet, smoking, and infections. Therefore, a single saliva sample is unlikely to be sufficient, and repeated measurements over time would be more appropriate to assess redox status.

At present, there are no published clinical studies reporting a direct correlation between salivary oxidative stress biomarkers and cardiac outcomes specifically related to sudden unexplained cardiac death. Most available clinical data on oxidative stress biomarkers come from other cardiovascular conditions, such as heart failure or coronary artery disease, and cannot be directly applied to SUCD. For this reason, no summary table of clinical SUCD studies was included, and this absence further highlights the need for future dedicated clinical research.

Therefore, salivary redox profiling should be regarded as a potential future research framework rather than a validated clinical screening strategy, pending confirmation in well-designed prospective cohort studies.

## 7. Conclusions

Salivary redox biomarkers represent potential, non-invasive indicators for exploring cardiac electrical vulnerability in apparently healthy young individuals. By reflecting systemic redox balance in an exploratory context, these biomarkers could help address an important research gap left by current clinical assessment approaches, which may not capture subtle molecular and redox-related disturbances preceding overt electrical or structural abnormalities. The integration of salivary redox profiling with clinical, electrocardiographic, genetic, and lifestyle data represents an emerging research direction in cardiology and in preventive cardiovascular research, rather than an established clinical application. Further collaborative, interdisciplinary research, together with the advances in biomarker technologies, may enable refinement of the “Salivary Redoxome” concept as a hypothesis-generating, research-oriented framework.

At present, this approach should be regarded as conceptual and investigational, pending confirmation in well-designed prospective studies. If supported by robust clinical and mechanistic evidence, salivary redox profiling may contribute to advancing the understanding of redox-related cardiac electrical vulnerability and to informing future research strategies in the study of sudden unexplained cardiac death in young individuals.

## 8. Statement of Contribution

Most studies on salivary oxidative stress biomarkers have focused on metabolic, neurodegenerative, and oral diseases, with limited investigation of their relevance to cardiac electrical vulnerability and no direct application to sudden unexplained cardiac death in young, apparently healthy individuals.

This review integrates current mechanistic and translational evidence linking redox imbalance to cardiac electrical instability and examines saliva as a non-invasive matrix for exploratory redox assessment. By synthesizing redox biology, arrhythmogenic mechanisms, and salivary biomarker research, it provides a hypothesis-generating framework to guide future experimental and clinical studies.

The value of this work lies in its integrative and research-oriented perspective, which clarifies conceptual links, methodological challenges, and key priorities for future validation, rather than proposing a clinical screening or predictive application.

## 9. Future Perspectives

Future research should focus on studying this conceptual framework through well-designed experimental and observational investigations to evaluate the biological relevance and reproducibility of salivary redox biomarkers in young, apparently healthy populations. The next phase should aim to establish reference ranges and analytical standardization for key oxidative and antioxidative markers, followed by exploratory correlation with electrophysiological, genetic, and lifestyle factors associated with cardiac electrical instability. Integration of these findings with methodological and analytical advances may support the development of standardized research frameworks suitable for large-scale prospective studies.

To move this project forward, it will require collaboration between several research centers, supported by coordinated academic and institutional efforts. Such coordinated efforts are important for evaluating the ‘Salivary Redoxome’ concept across diverse populations and for determining how it may complement existing molecular and physiological approaches to studying cardiac electrical vulnerability.

## Figures and Tables

**Table 1:** Salivary redox biomarkers explored in cardiovascular and arrhythmogenic research.

Biomarker	Mechanistic Relevance to Arrhythmogenic Vulnerability	Evidence in Cardiovascular RESEARCH	Key Limitations
Superoxide dismutase (SOD)	First-line antioxidant defense; regulates superoxide-mediated ion-channel dysfunction	Altered levels reported in cardiac arrhythmias, heart failure, and oxidative stress–related conditions	Influenced by exercise, diet, circadian rhythm
Catalase (CAT)	Detoxifies hydrogen peroxide; protects calcium-handling proteins	Reduced activity linked to myocardial oxidative injury	Limited salivary-specific cardiac studies
Glutathione peroxidase (GPx)	Maintains redox balance; prevents lipid peroxidation	Associated with arrhythmogenic vulnerability and mitochondrial dysfunction	Enzyme stability varies pre-analytically
Malondialdehyde (MDA)	Marker of lipid peroxidation and oxidative damage	Elevated in cardiovascular diseases and electrical instability	Not disease-specific
Total antioxidant capacity (TAC)	Integrated measure of enzymatic and non-enzymatic defenses	Reflects systemic oxidative balance in cardiac and metabolic disorders	Influenced by nutrition and lifestyle

Data summarized from previously published studies [7,11,24].

## Data Availability

This article is a review of previously published studies. No new data were generated or analyzed during the current study.

## Abbreviations

The following abbreviations are used in this manuscript:

ATP	Adenosine Triphosphate
CAD	Coronary Artery Disease
CAT	Catalase
CaMKII	Calcium/Calmodulin-Dependent Protein Kinase II
Ca^2+^	Calcium ion
ECG	Electrocardiogram / Electrocardiography
ELISA	Enzyme-Linked Immunosorbent Assay
GPx	Glutathione Peroxidase
HF	Heart Failure
K^+^	Potassium ion
MDA	Malondialdehyde
Na^+^	Sodium ion
NADPH	Nicotinamide Adenine Dinucleotide Phosphate
OMICS	Comprehensive molecular profiling methods
RNS	Reactive Nitrogen Species
ROS	Reactive Oxygen Species
SUDY	Sudden Unexpected Death in Young
SCD	Sudden Cardiac Death
SOD	Superoxide Dismutase
SUCD	Sudden Unexplained Cardiac Death
TAC	Total Antioxidant Capacity
VF	Ventricular Fibrillation
VT	Ventricular Tachycardia
